# Codon and Reverse Codon: A Theoretical Approach to Reinterpret the Genetic Code Table

**DOI:** 10.7759/cureus.48598

**Published:** 2023-11-10

**Authors:** Nicola Serra, Paola Di Carlo

**Affiliations:** 1 Department of Public Health, University Federico II of Naples, Naples, ITA; 2 Department of Infectious Diseases, Policlinico Paolo Giaccone University Hospital, Palermo, ITA; 3 Department of Health Promotion, Maternal-Childhood, Internal Medicine of Excellence "G. D'Alessandro,” Promise, University of Palermo, Palermo, ITA

**Keywords:** d-amino acids, l-amino acid, reverse codon, codon, amino acid, nucleotide, genetic code table

## Abstract

The genetic code table represents a fundamental scheme to translate a genetic code into a sequence of amino acids and, therefore, the possibility of operating the synthesis of all the proteins necessary for the life of organisms. Unfortunately, the various biological mechanisms are not fully clear. Hence, in this report, we analyzed the genetic code table and the amino acids codified by codons with an original theoretical and statistical approach based on the concept of permutations. We found an interesting reinterpretation of many codons, as reverse codons, which could help clarify some as-yet-unknown aspects in the field of protein folding.

## Introduction

In the early 1940s, even though it was known that genes determine hereditary characteristics, and they are located on the chromosomes, their exact chemical nature was unknown. Since it was known that chromosomes consisted mainly of deoxyribonucleic acid (DNA) and proteins, the repository of biological information must have been one of these two molecules. Most researchers believed that the genetic material was represented by proteins and that DNA, made up of only four components, (nucleotides) was too simple to justify the variety and richness of biological information. However, this assumption turned out to be wrong. The decisive confirmation that DNA is the repository of genetic information was provided by Hershey et al. [1]. They showed that the bacteriophages injected their DNA genome into the cytoplasm of the host bacterium to introduce their hereditary material. In the early 1950s, James Watson and Francis Crick [2], analyzing all available data, identified the three-dimensional structure of DNA, proposing the well-known double helix model.

The genetic code is the system by which the information encoded in the DNA defines the synthesis of all the proteins necessary for the life of organisms. Its language is based on a molecular "alphabet" represented by the DNA nucleotide sequence, where every nucleotide consists of three components: a nitrogenous base, a sugar with five carbon atoms, which in DNA is the deoxyribose, and a phosphate group. The genetic code is characterized by four "letters", which represent four different nitrogenous bases or nucleotides, two purine bases: adenine (A), guanine (G), and two pyrimidine bases: cytosine (C) and thymine (T). The order in the sequential arrangement of the nucleotides constitutes the genetic information, which is translated via the genetic code into the corresponding amino acids [3,4]. Using groups of three nucleotides (codon), it is possible to obtain 4^3^ = 64 different codons, sufficient to encode the 20 amino acids that characterize the language on which a protein is defined. Each amino acid is a group of organic molecules that consists of a basic amino group (―NH2), an acidic carboxyl group (―COOH), and an organic R group (or side chain) that is unique to each amino acid and defines its characteristics. More than 200 amino acids are known, but only 20 of them are used to build the proteins of living organisms (on Earth). According to the literature, the 20 amino acids are indicated as follows: alanine (A or ALA), arginine (R or ARG), asparagine (N or ASN), aspartic acid (D or ASP), cysteine (C or CYS), glutamine (Q or GLN), glutamic acid (E or GLU), glycine (G or GLY), histidine (H or HIS), isoleucine (I or ILE), leucine (L or LEU), lysine (K or LYS), methionine (M or MET), phenylalanine (F or PHE), proline (P or PRO), serine (S or SER), threonine (T or THR), tryptophan (W or TRP), tyrosine (Y or TYR), valine (V or VAL).

The genetic code table has several interesting characteristics. It is unambiguous, i.e., each codon always specifies a well-defined amino acid; it is redundant, i.e., almost all amino acids are specified by more than one codon; it is universal, i.e., it is valid for all organisms, with the exception represented by mitochondrial DNA, whose code is different from the nuclear one. Moreover, it is characterized by a start signal represented by the ATG codon and three stop codons (TAA, TAG, and TGA). A complex characteristic of the genetic code table is redundancy. For this reason, many authors agree that it needs to be improved and have proposed its reinterpretation by applying various methods based on physical and mathematical approaches [5-11].

The objective of this study is to reinterpret the genetic code table with a purely and simply theoretical approach based on the statistical concept of permutations, by introducing a new concept of “reverse codon (r-codon)” to reduce the redundancy effect and thereby improve the genetic code table.

## Technical report

**Figure 1:**
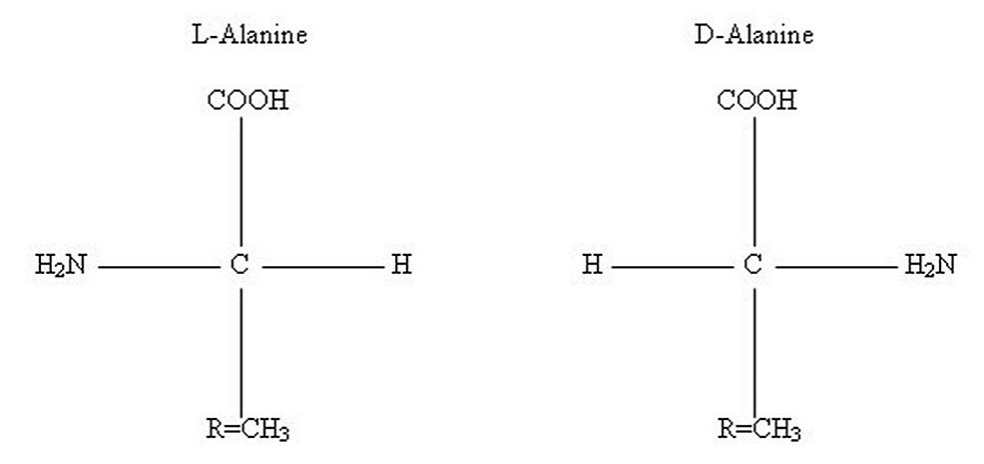
Stereochemical arrangement for L- and D-alanine

Of the 20 common amino acids in the human body that build our proteins, each of them (except for glycine) occurs in two isomeric forms, namely L-amino acids - we will call them left-handed amino acids - and D-amino acids - we will call those the right-handed amino acids. L- and D-amino acids have different orientations of the four substituents attached to the amino acid’s central carbon atom, also known as the chiral carbon or alpha carbon. The four substituents are represented by a single hydrogen atom (H); a carboxyl group (or COOH group); an amine group (or NH2 group); and the distinguishing R group, which mostly differentiates one amino acid from another. L- and D-amino acids have the same molecular formula but two different structural formulas and are known as non-superimposed mirror images; so the arrangement of the atoms is also different. This implies different physical and chemical properties, due to the different bonds of the elements that make up the molecule. Particularly, L-amino acid has a structure rearranged in a “reverse way” than D-amino acid, according to the Fisher classification [12,13]. Figure 1 shows the stereochemical arrangement for L- and D-alanine.

For a long time, it was believed that for some reason, the amino acids that make up the proteins in our body were all L-amino acids. Nevertheless, in living organisms, not only free forms of D-amino acids but also several D-amino acids containing peptides (DAACPs) have been isolated [14,15]. Among these DAACPs, many are biologically active while their counterpart peptides synthesized from only L-amino acids are either totally inactive or have minimum activity [16].

Below, we will show how the concept of r-codon introduced in this paper can be linked to L- or D-amino acid. Table 1 shows the standard genetic code table, where all possible codons and the corresponding amino acids are presented.

**Table 1:** Standard genetic code table where each codon is associated with one or more amino acids

	Second letter				
First letter	T	C	A	G	Third letter
T	TTT=Phe	TCT=Ser	TAT=Tyr	TGT=Cys	T
C	CTT=Leu	CCT=Pro	CAT=His	CGT=Arg	T
A	ATT=Ile	ACT=Thr	AAT=Asn	AGT=Ser	T
G	GTT=Val	GCT=Ala	GAT=Asp	GGT=Gly	T
T	TTC=Phe	TCC=Ser	TAC=Tyr	TGC=Cys	C
C	CTC=Leu	CCC=Pro	CAC=His	CGC=Arg	C
A	ATC=Ile	ACC=Thr	AAC=Asn	AGC=Ser	C
G	GTC=Val	GCC=Ala	GAC=Asp	GGC=Gly	C
T	TTA=Leu	TCA=Ser	TAA=stop	TGA=stop	A
C	CTA=Leu	CCA=Pro	CAA=Gln	CGA=Arg	A
A	ATA=Ile	ACA=Thr	AAA=Lys	AGA=Arg	A
G	GTA=Val	GCA=Ala	GAA=Glu	GGA=Gly	A
T	TTG=Leu	TCG=Ser	TAG=stop	TGG=Trp	G
C	CTG=Leu	CCG=Pro	CAG=Gln	CGG=Arg	G
A	ATG=Met(start)	ACG=Thr	AAG=Lys	AGG=Arg	G
G	GTG=Val	GCG=Ala	GAG=Glu	GGG=Gly	G

Standard genetic code, as described in Table 1, shows many codons that differ only by the third nucleotide (synonymous codons). The presence of synonymous codons is generally a positive aspect. It constitutes a defense against mutations because if a mutation occurs, it will lower the probability of inserting an incorrect amino acid in protein synthesis. In addition, three codons do not encode any amino acids (TAA, TAG, TGA). They represent the stop signal of the protein chain, while all the others encode amino acids. Particularly, in Table 1, we identify four codons represented by the same nucleotide called periodical codons [17]: AAA = Lys, TTT = Phe, CCC = Pro, GGG = Gly, twelve palindromic codons: ATA = Ile, ACA = Thr, AGA = Arg, CAC = His, CTC = Leu, CGC = Arg, TAT = Tyr, TCT = Ser, TGT = Cys, GAG = Glu, GTG = Val, GCG = Ala, three codons TAA, TAG and TGA representing the stop signal of the protein chain, and all the remaining 45 codons represent all others possible permutations of three of the four nucleotides which identify specific amino acids.

**Figure 2:**
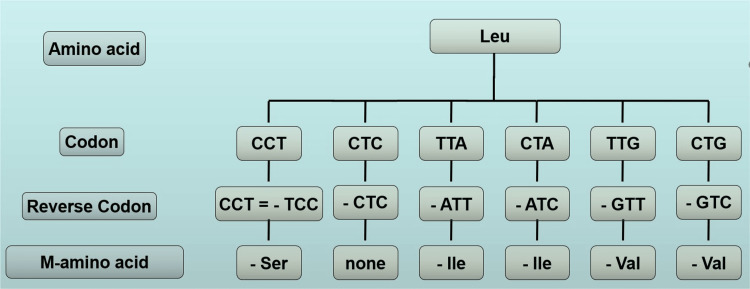
Codons, r-codons, and M-amino acids linked to leucine

In this paper, we only consider 45 codons, i.e., three codons of stop signal and 42 codons represented by all possible permutations except the palindromic, the periodical codons, and the three codons GGT, GGC, and GGA which code for the glycine amino acid. For these codons, a reinterpretation is possible. For example, we consider among the aforementioned 45 codons, the codon TTA = Leu; this codon represents an ordered triplet of nucleotides. Therefore, it results in TTA(Leu) ≠ ATT(Ile), according to Table 1. If we consider this codon as a box, the ATT codon could be represented by the same box related to the TTA codon but inserted in the protein chain in reverse mode. In other words, the ATT codon could be considered as a box functioning in the reverse way in comparison to the TTA codon. Therefore, the TTA codon (Leu) could be reinterpreted as an amino acid Ile in reverse position, i.e., in mirror position in a protein chain (-Ile) and indicated as M-Ile, which represents the L- or D-Ile depending on the stereochemical arrangement of Leu (D- or L-Leu, respectively). In Figure 2, we show all codons associated with the Leu amino acid, the r-codons, and the M-amino acids.

In Table 2, we present a possible description of the genetic code table reinterpreted according to the idea of r-codon and M-amino acids. M-amino acids are reported with a minus sign ("-").

**Table 2:** Theoretical reinterpretation of the standard genetic code table

	Second letter				
First letter	T	C	A	G	Third letter
T	TTT=Phe	TCT=Ser	TAT=TYR	TGT=Cys	T
C	CTT=Leu	CCT=Pro	CAT=His	CGT=Arg	T
A	ATT=Ile	ACT=Thr	AAT=Asn	AGT=Ser	T
G	GTT=Val	GCT=Ala	GAT=Asp	GGT=Gly	T
T	TTC=Phe(-Leu)	TCC=Ser(-Pro)	TAC=Tyr(-His)	TGC=Cys(-Arg)	C
C	CTC=Leu	CCC=Pro	CAC=His	CGC=Arg	C
A	ATC=Ile	ACC=Thr	AAC=Asn	AGC=Ser	C
G	GTC=Val	GCC=Ala	GAC=Asp	GGC=Gly	C
T	TTA=Leu(-Ile)	TCA=Ser(-Thr)	TAA=stop(-Asn)	TGA=stop(-Ser)	A
C	CTA=Leu(-Ile)	CCA=Pro(-Thr)	CAA=Gln(-Asn)	CGA=ARG(-Ser)	A
A	ATA=Ile	ACA=Thr	AAA=Lys	AGA=Arg	A
G	GTA=Val	GCA=Ala	GAA=Glu	GGA=Gly	A
T	TTG=Leu(-Val)	TCG=Ser(-Ala)	TAG=stop(-Asp)	TGG=Trp	G
C	CTG=Leu(-Val)	CCG=Pro(-Ala)	CAG=GLN(-Asp)	CGG=Arg	G
A	ATG=Met(start) (-Val)	ACG=Thr(-Ala)	AAG=LYS(-Glu)	AGG=Arg	G
G	GTG=Val	GCG=Ala	GAG=Glu	GGG=Gly	G

In Table 2, for each column, it is possible to observe three palindromic codons, one periodical codon, six “normal codons” and six r-codons except for the G column (three r-codons).

Finally, we performed computer simulations to evaluate whether the structure arrangement was stable after changing one amino acid to an M-amino acid. For this purpose, we used the Avogadro software ver. 1.2.0. It is an advanced molecular editor designed for cross-platform use in computational chemistry, molecular modeling, bioinformatics, materials science, and related areas [18]. Also, we considered the hormone of human insulin (pdb3i40, PDB DOI: https://doi.org/10.2210/pdb3i40/pdb) to perform the simulations. This hormone structure was selected, because it is composed of two helixes structure only, and characterized by 21 and 30 amino acids, respectively. For simplicity, we considered only the helix composed of 21 amino acids: Gly-Ile-Val-Glu-Gln-Cys-Cys-Thr-Ser-Ile-Cys-Ser-*Leu*-Tyr-Gln-Leu-Glu-Asn-Tyr-Cys-Asn.

**Figure 3:**
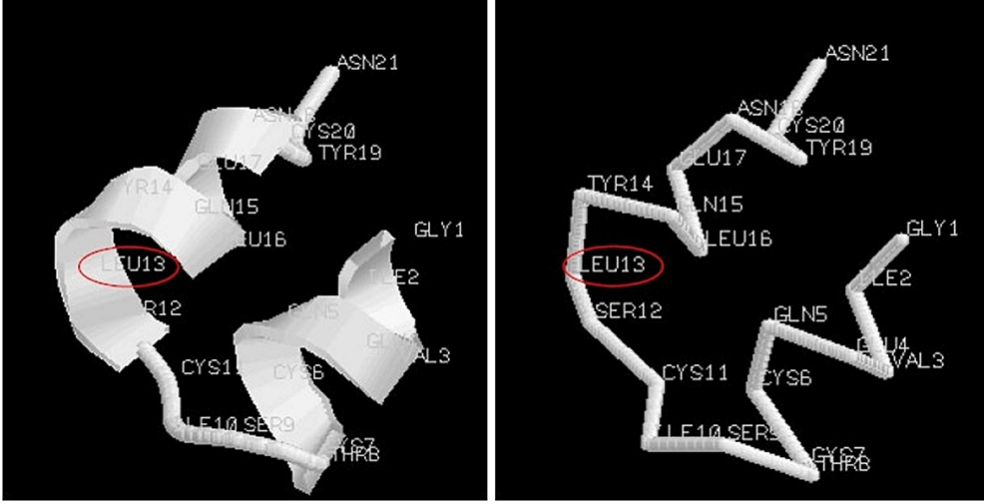
Real structure shown with Avogadro software: (a) cartoons; (b) backbone

**Figure 4:**
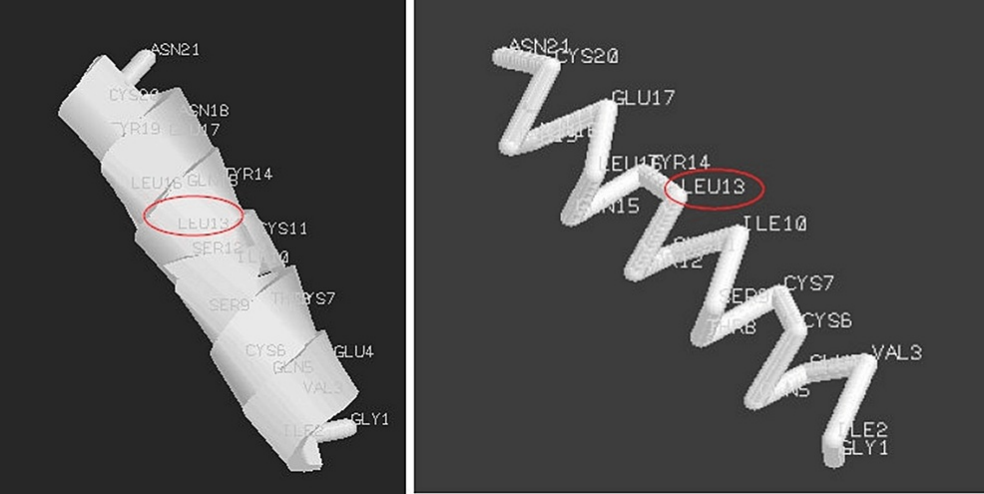
Real structure simulated with Avogadro software: (a) cartoons; (b) backbone

**Figure 5:**
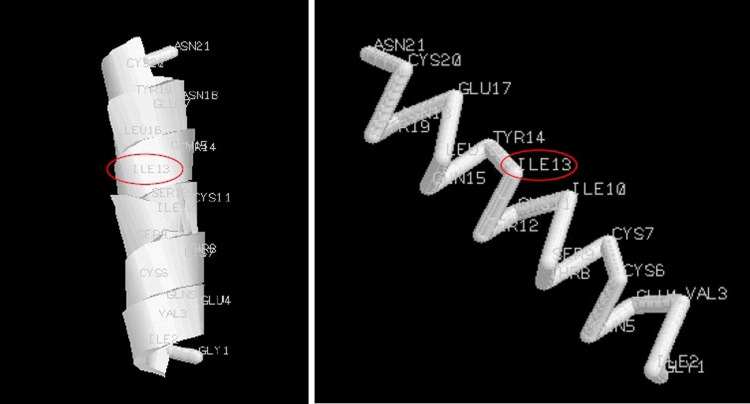
Theoretical structure simulated with Avogadro software where L-Leu was replaced with D-Ile: (a) cartoons; (b) backbone

We considered the 13th amino acid L-Leu (shown between two asterisks in the previous amino acidic sequence and marked with a red circle in the following figures) and replaced it with D-Ile according to our theory. Particularly, we considered the 13th L-Leu amino acid, because it was positioned approximately in the center of the structure. In fact, we considered it more interesting to observe a central amino acid than an amino acid positioned more towards the external areas of the helix. In Figures 3-5, we respectively show the real structure (helix structure from the PDB file), the simulated real structure using only the original amino acids of the helix sequence, and the simulated theoretical structure where we changed the 13th amino acid in the helix sequence. In particular, the real 3D structure of the helix was obtained by the atomic coordinates of the PDB file (Figure 3), while the real and theoretical simulated structures (Figures 4, 5) were obtained by considering the spatial composition of amino acid by amino acid.

In addition, the properties of the helix for the real structure, the simulated real structure, and the simulated theoretical structure are reported below.

Real structure: molecular weight = 2401.84 g/mol, estimated dipole moment = 93.95, chemical formula = C_99_H_171_N_25_O_35_S_4_, number of atoms = 318, number of bonds = 333, number of residuals = 21. 

Real structure simulated: molecular weight = 2383.71 g/mol, estimated dipole moment = 111.62, chemical formula = C_99_H_153_N_25_O_35_S_4_, number of atoms = 318, number of bonds = 317, number of residuals = 21.

Theoretical structure simulated: molecular weight = 2383.70, estimated dipole moment = 111.60, chemical formula = C_99_H_153_N_25_O_35_S_4_, number of atoms = 318, number of bonds = 317, number of residuals = 21.

It can be observed that the simulated real structure and the simulated theoretical structure showed similar properties.

## Discussion

Many authors have claimed that there is a need to optimize the genetic code table, in the scientific literature. Different approaches have been used for this goal, such as that by Doig [9], who proposed to improve the efficiency of the genetic code by varying the codon length, while Berleant et al. [5] presented a novel superposition of the BLOSUM62 matrix and an allowed point mutation matrix. This superposition depicts an important aspect of the true genetic code: its ability to tolerate mutations and mistranslations. Karasev [19] investigated the periodic properties of triplets in the Canonical Table of the Genetic Code (CTGC). A statistical approach was proposed by Štambuk and Konjevoda [20], where the authors investigated all possible alternatives for the encoding of four nucleobases and related amino acid properties, concerning (1) the three-dimensional codon arrangement or positions, and (2) all possible 24 hierarchical base partitions at each codon base, determining the amino acid scores on their codon hierarchy description.

Wilhelm and Nikolajewa [21] presented a new classification scheme of the genetic code based on a binary representation of the purines and pyrimidines. The scheme reveals known patterns more clearly than the common one; for instance, the classification of strong, mixed, and weak codons and the ordering of codon families, showing new patterns that have not been described before.

The approach described by Wong [22] was very interesting. Specifically, he proposed that the structure of the genetic code was determined by the sequence of evolutionary emergence of new amino acids within the primordial biochemical system. Biro et al. [23] put forward a periodic table of codons where the codons were in regular locations. The table was composed of four fields (16 places in each) one with each of the four nucleotides (A, U, G, and C) in the central codon position, and where AAA (lysine), UUU (phenylalanine), GGG (glycine), and CCC (proline) were placed into the corners of the fields as the main codons (and amino acids) of the fields. Kurić [24] proposed a different approach, based on the investigation of the biochemical basis of genetic processes through the digital mechanism of nucleic acid and protein biosynthesis and the evolution of biomacromolecules. Particularly, the biochemical evolution of genetic language has been analyzed by the application of cybernetic methods, information theory, and system theory, respectively.

On the other hand, new ideas about the structure of the genetic code table have been proposed through physical and mathematical approaches, using, e.g., concepts of symmetry [25-27] and group theory [28]. Regarding the symmetry of the genetic code table, recently, Rosandić and Paar [29] formulated the new ideal symmetry genetic code table, which reflects a unique fundamental physicochemical purine-pyrimidine symmetry net, for all of over 30 known variations of nuclear and mitochondrial genetic codes.

The reinterpretation of the genetic code table proposed in this study is conceptually different from that in all previously published studies. Particularly, it is based on the concept of r-codon, different from the concept of anticodon, which represents a triplet of nucleic acids located on the tRNA molecule and complementary to a specific codon, on the mRNA. In this way, it could be possible to interpret many codons from another perspective, thereby reducing the redundancy effect and improving the genetic code table. Regarding the codon ATT that was used to explain the concept of the r-codon in the previous section, we have observed that TTA(Leu) ≠ ATT(Ile). The choice to assign TTA = -Ile(M-Ile) in the genetic code table is arbitrary. In fact, in an equivalent way, we could assign ATT= -Leu (M-Leu), based on our idea. This choice should be made by considering chemical, physical, and structural characteristics. Hence, we can also reinterpret the stop signal codons TAA, TAG, and TGA as amino acids. According to our idea, they could be interpreted by the following M-amino acids: -Asn (M-Asn), -Asp (M-Asp), and -Ser (M-Ser), explaining in an original way how the stop signal codons also encode amino acids. In this case, each r-codon corresponds to only one stop codon, i.e., there is a univocal correspondence between the r-codons and the stop codons; therefore, the M-amino acid associated with the corresponding stop codon is unique.

From computer simulations, it can be observed that the simulated structures (Figures 4, 5) are similar, such as in their physiochemical properties. Particularly, Figure 5, where the amino acid L-Leu has been replaced with D-Ile, shows no gap in the structure. These simulations would seem to confirm the idea that the replacement of an amino acid with its M-amino acid (L- or D- D-amino acid) is possible.

## Conclusions

This study is based on a theoretical and statistical approach that introduces a new point of view in terms of reinterpreting the genetic code table. The authors focused their attention on the method, introducing a new concept of r-codon and showing how it is linked to M-amino acid, and how it could be particularly useful in reducing the redundancy of the genetic code table. Generally, an M-amino acid associated with r-codon is represented by a D-amino acid. This depends on a higher frequency of L-amino acids in protein sequences. Particularly, we observe that if there is a codon associated not with an L-amino acid but with a D-amino acid, its r-codon will be associated with the corresponding L-amino acid; e.g., ATT(D-Ile) implicate -ATT(L-Ile) = TTA (L-Leu), and hence, in an equivalent way, we could suggest that ATT(L-Ile) implicate -ATT(D-Ile) = TTA (D-Leu).

In conclusion, the interesting reinterpretation of a part of the codons in the genetic code table as r-codon blends well with the concepts of L- and D-amino acid, thereby reducing table redundancy and paving the way for potential and intriguing applications on the use of the r-codon in the protein structures analysis.
